# Revolutionizing precision oncology: the role of artificial intelligence in personalized pediatric cancer care

**DOI:** 10.3389/fmed.2025.1555893

**Published:** 2025-05-19

**Authors:** Hasan Hashem, Iyad Sultan

**Affiliations:** ^1^Department of Pediatrics, Division of Pediatric Hematology-Oncology, King Hussein Cancer Center (KHCC), Amman, Jordan; ^2^Pediatric Bone Marrow Transplantation and Stem Cell Therapy Service, King Hussein Cancer Center (KHCC), Amman, Jordan; ^3^Artificial Intelligence Office, King Hussein Cancer Center (KHCC), Amman, Jordan; ^4^Department of Pediatrics, Faculty of Medicine, University of Jordan, Amman, Jordan

**Keywords:** artificial intelligence, precision medicine, personalized medicine, pediatric cancer, pediatric oncology, machine learning, deep learning

## Abstract

Artificial intelligence (AI) has recently garnered significant public attention. Among the various fields where AI can be applied, medicine stands out as one with immense potential. In particular, AI is transforming precision oncology by providing innovative approaches to customize cancer treatments for individual patients. This article examines the latest developments in AI-powered tools designed to improve cancer diagnosis accuracy and predict treatment outcomes. The integration of AI into precision oncology is transforming cancer care by enabling more personalized and effective treatments, minimizing treatment-related toxicities, and enhancing patient survival rates. As AI advances, it will be pivotal in developing more targeted and successful cancer therapies. The field is still in its early stages, and future progress will benefit from establishing standards and guidelines to promote rigorous methodological design and uphold ethical principles. This research highlights the transformative potential of AI in addressing the challenges posed by cancer heterogeneity.

## 1 Introduction

Pediatric cancers constitute a critical health challenge worldwide. Each year, approximately 400,000 children and adolescents (ages 0–19) are diagnosed with cancer globally, making it a leading cause of death in this age group (1). Leukemia, brain tumors, lymphomas, neuroblastoma, and Wilms tumors are among the most common malignancies encountered in pediatric populations (1). Tragically, more than 100,000 pediatric cancer deaths occur annually worldwide, underscoring significant disparities in survival rates, with approximately 80% survival in high-income countries compared to less than 30% in lower-resource settings (2). These stark statistics highlight the urgent need for advancements in precision oncology to improve early detection, accurate diagnosis, and effective personalized treatment strategies in pediatric cancer care.

Artificial intelligence (AI) algorithms have been applied in numerous medical tasks efficiently and accurately. AI algorithms are increasingly being integrated into pediatric oncology, offering promising advancements in diagnosing, treating, and managing childhood cancers. This integration aims to enhance precision oncology, which focuses on tailoring treatment to the individual characteristics of each patient and their disease (3, 4)

AI strategies can tackle enormous amounts of original data in a short time to solve complex tasks with high accuracy. AI is mainly implemented with machine learning (ML) and deep learning (DL) working principles. AI algorithms capture data, identify patterns, and provide decisions and predictions about actual real-world events. AI can help address the lack of objectivity and universality in expert systems. However, it can also run the risk of overfitting training data, thus often a tradeoff between accuracy and intelligibility (5).

**FIGURE 1:**
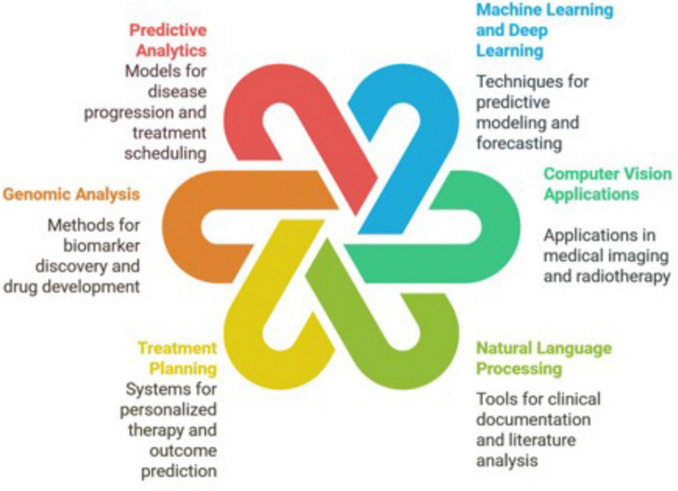
The interaction of artificial intelligence with the field of oncology is represented under six major domains: machine/deep learning, computer vision, natural language processing, predictive analytics, genomic analysis, and treatment planning.

While the field of artificial intelligence (AI) in oncology is rapidly evolving, we present its interaction with the field under six major domains (Figure 1): Machine/Deep Learning, Computer Vision, Natural Language Processing, Predictive Analytics, Genomic Analysis, and Treatment Planning (6).

Although the development of these AI systems relies heavily on intricate mathematical models and robust datasets for training and inference, healthcare professionals are generally less concerned with the technical underpinnings. Instead, they prioritize the seamless integration of AI into their workflows. The goal is to streamline administrative tasks, reduce errors, and free up time to focus on patient care. Furthermore, AI should minimize bias in clinical decision-making without introducing unnecessary complexity into care delivery. This approach ensures that AI as a tool to enhance the quality of care rather than complicating it.

## 2 Methods

This review paper utilizes a deep research methodology to provide a comprehensive overview of the role of artificial intelligence (AI) in personalized pediatric cancer care. Our research involved an iterative and in-depth exploration of information from diverse sources to gain a thorough understanding of the topic. The research process involved the following steps:

1. *Information gathering*: A wide range of sources were consulted to gather information on AI in pediatric precision oncology, including research articles, clinical trial databases, medical journals, and reputable online resources.
2. *Synthesis and analysis*: The gathered information was carefully synthesized and analyzed to identify key themes, trends, and challenges related to the application of AI in pediatric cancer care.
3. *Iterative refinement:* The research process involved an iterative information refinement, where initial findings were further explored and validated through additional research and analysis.
4. *Critical evaluation:* The information was critically evaluated to ensure accuracy, relevance, and reliability.

This profound research methodology allowed for a comprehensive and nuanced understanding of the role of AI in personalized pediatric cancer care, enabling the identification of key applications, challenges, ethical considerations, and future directions. The following tools were also used to enhance this search and edit this paper: Google Gemini deep research, OpenAI ChatGPT, Anthropic Claude, Perplexity, and napkin.ai.

## 3 Introduction to AI

Artificial Intelligence (AI) is a multidisciplinary field that combines mathematics, computer science, and data analysis to develop systems capable of performing tasks that typically require human intelligence, such as learning, reasoning, and problem-solving. In medicine, AI encompasses a variety of algorithms and models designed to interpret complex medical data, enhance diagnostic accuracy, and improve patient care (7).

One of the foundational techniques in AI is regression analysis, including linear and logistic regression. These statistical methods enable the prediction of outcomes based on input variables known as features. Linear regression predicts continuous outcomes, while logistic regression is used for binary outcomes, such as the presence or absence of a disease. These models are integral to medical research and practice, forming the basis for many clinical decision-making processes (8).

As AI has advanced, more sophisticated algorithms have been developed to handle complex datasets. Clustering algorithms, like k-nearest neighbors, group similar data points, aiding pattern recognition within patient populations. Dimensionality reduction techniques, such as Principal Component Analysis (PCA) and Singular Value Decomposition (SVD), simplify large datasets by reducing the number of variables, making it easier to visualize and interpret data without significant loss of information (9).

Decision trees are widely used in predictive modeling for their ability to model decisions and their possible consequences. These have evolved into more robust methods like random forests and gradient boosting machines (e.g., XGBoost), which improve predictive performance by combining multiple decision trees to reduce overfitting and enhance accuracy. These ensemble methods are particularly effective in analyzing tabular medical data, such as electronic health records, to predict patient outcomes and inform treatment strategies (10).

The development of neural networks has been pivotal in AI, leading to the emergence of deep learning. These networks, inspired by the human brain’s architecture, modeling can model complex, non-linear relationships in data. In medical imaging, Convolutional Neural Networks (CNNs) have revolutionized computer vision tasks by enabling machines to accuracy accurately interpret visual data. CNNs are extensively used in analyzing radiological images, assisting in the detection and diagnosis of conditions such as tumors and fractures (11).

The introduction of transformer architectures has further advanced AI capabilities, particularly in natural language processing. Transformers utilize attention mechanisms to process and generate human language, facilitating the development of large language models that can comprehend and produce text with remarkable proficiency. These models have also been adapted for multimodal applications, integrating visual and textual data to enhance diagnostic tools and decision support systems in healthcare.

Additionally, AI plays a significant role in time series forecasting, which involves analyzing time-ordered data to predict future events. In healthcare, this is essential for anticipating disease outbreaks, managing hospital resources, and monitoring patient vital signs to detect early warning signs of deterioration.

Integrating AI into medicine holds great promise for enhancing diagnostic accuracy, personalizing treatment plans, and improving overall patient outcomes. As AI technologies continue to evolve, their applications in healthcare are expected to expand, offering innovative solutions to complex medical challenges.

### 3.1 Machine/deep learning

AI, encompassing Machine Learning (ML) and Deep Learning (DL), has emerged as a transformative tool in pediatric oncology. ML is a more generic term that includes many AI algorithms, including neural networks. These models learn by analyzing a large set of data. ML algorithms analyze large datasets to identify patterns, make predictions, and support decision-making. Their scope can be extended to analyze text pre-processed to become a “bag of words.” DL is a more precise term that includes deep neural networks with multiple hidden layers. Technologies that advanced basic neural networks to more advanced deep networks included computational and mathematical advancement. Nevertheless, these networks remain computationally costly when used to process natural language or large images. The invention of transformers and Convolutional Neural Networks (CNNs) solved these problems and offered enhanced accuracy and efficiency with minimal pre-processing.

Training machine learning (ML) and deep learning (DL) models rely on constructing a large dataset containing accurate dependent variables, such as disease presence, side effect development, or mortality, along with carefully curated explanatory variables, also known as features. The correct data, referred to as the ground truth, is typically divided into training and testing subsets. These subsets are used to train the model and validate its performance. Metrics used to evaluate training accuracy include accuracy, F1 score, and AUC. Table 1 provides a summary of these metrics.

**TABLE 1:** Common metrics for evaluating ML/DL model accuracy.

Metric	Description
Accuracy	Measures the proportion of correct predictions among the total predictions.
F1 score	The harmonic mean of precision and recall, useful for imbalanced datasets.
AUC (area under curve)	Represents the ability of the model to distinguish between classes.
Precision	Measures the proportion of true positive predictions out of all positive predictions.
Recall	Measures the proportion of true positive predictions out of all actual positives.

A study analyzed data from 1,433 chemotherapy cycles involving 437 children diagnosed with Wilms’ tumor. The extreme gradient boosting (XGB) model demonstrated the best predictive efficiency, achieving an area under the receiver operating characteristic curve (AUROC) of 0.981 in the training set and 0.896 in the test set. The model’s ability to accurately predict grade ≥ 2 CIM allows for early preventive management strategies, thereby optimizing patient wellbeing during treatment (12).

Despite these advancements, challenges persist. The rarity of pediatric cancers and their molecular heterogeneity limit the availability of large, standardized datasets necessary for training robust AI models. This scarcity impedes the development of universally applicable algorithms. Moreover, ethical considerations, including patient privacy and data security, are paramount, especially given the sensitivity of health information pertaining to children (13).

### 3.2 Computer vision

Computer vision, a branch of artificial intelligence, aims to empower machines to understand and analyze visual information from images and videos. Convolutional neural networks (CNNs) achieve this by learning from small groups of pixels called kernels. This breakthrough allows neural networks to focus on key details in an image, simplifying the learning process and enhancing their ability to interpret visual data effectively. Segmentation refers to the process of delineating structures by training special CNN to recognize the borders of essential parts of the image (e.g., pulmonary nodule, pituitary gland, etc.).

For example, AI algorithms have been developed to classify soft-tissue and bone tumors on radiological images, achieving accuracy comparable to experienced specialists. In brain tumors, ML models have improved diagnostic accuracy by analyzing MRI sequences, particularly in challenging cases where tumor types overlap (14). Additionally, CNN models have been investigated for detecting pulmonary nodules in patients with osteosarcoma, with results comparable to medical doctors (92.3 vs. 90.8%) (15, 16).

However, the development of AI algorithms for pediatric tumor segmentation faces challenges due to heterogeneous imaging protocols, variations in patient anatomy, and the limited availability of multi-institutional data (17). Addressing these challenges requires collaborative efforts to create large, standardized datasets and to develop algorithms specifically for pediatric populations (18).

### 3.3 Natural language processing

Natural Language Processing (NLP), a subfield of artificial intelligence, focuses on enabling machines to comprehend, interpret, and generate human language. The advent of transformers, driven by attention mechanisms, has significantly advanced the capability of neural networks to understand natural language. This process relies on creating numerical representations of words, known as embeddings, which are derived from analyzing text and grasping the semantic meaning of tokens (subword units). Additionally, the positional information of each word within a sentence is encoded using positional embeddings. These numerical vectors traverse through multiple layers of neural networks, interacting within specialized matrices designed to learn and represent the contextual meaning of words. This process is elegantly articulated through sophisticated mathematical formulations (4).

In pediatric oncology, NLP has been applied to extract valuable insights from unstructured clinical data, such as electronic health records (EHRs), medical literature, and patient narratives.

NLP tools can assist in summarizing patient histories, identifying relevant clinical information, and supporting decision-making processes. For instance, NLP algorithms can analyze clinical notes to identify patients eligible for clinical trials, facilitating patient recruitment and personalized treatment strategies. Additionally, NLP can aid in mining medical literature for new treatment strategies and biomarkers, supporting evidence-based practice.

One notable example of utilizing NLP in pediatric oncology is ExtractEHR, an innovative software tool designed to extract and curate data from electronic health records (EHRs). Originally developed to enhance adverse event reporting in clinical trials, ExtractEHR has expanded its capabilities to support the creation of multisite pediatric cancer data sets, facilitating diverse research applications. This tool has been successfully implemented in institutions like the Children’s Hospital of Philadelphia and Texas Children’s Hospital. It enables the extraction and cleaning of structured and unstructured data, including laboratory results and clinician notes, through its companion modules CleanEHR and GradeEHR. These capabilities have been used in various research contexts, such as studying treatment-related hepatotoxicity and acute kidney injury in leukemia patients, identifying complications like typhlitis using automated algorithms, and streamlining data collection for clinical trials and registries like SEER. By overcoming traditional data silos and enhancing the granularity of data analysis, ExtractEHR represents a significant advancement in pediatric oncology research, offering cost-effective and scalable solutions to support real-world data integration and patient care improvements (19).

### 3.4 NLP in clinical trial recruitment

Recently, investigators from multiple institutions developed and evaluated a clinical trial matching system called PRISM, utilizing a custom-tuned LLM called OncoLLM1. OncoLLM demonstrated superior performance compared to other models like GPT-3.5 Turbo and matched the performance of qualified medical doctors for clinical trial matching1. The model achieved 63% accuracy in question answering related to patient eligibility, which increased to 66% when ambiguous inputs were excluded 34. OncoLLM also performed well in concept-wise accuracy, outperforming GPT-4 in biomarkers. In ranking trials, OncoLLM ranked ground truth trials in the top three 65.3% of the time in patient-centric searches and achieved an NDCG score of 0.68 in trial-centric searches. The model met 62% of the criteria on average for ground truth trials, an accuracy of 66.7% for trials ranked in the top three 910. Furthermore, OncoLLM also produced fewer “N/A” responses than GPT-3.510. A key benefit is the significantly lower cost of OncoLLM, approximately $0.17 per patient-trial pair, compared to $6.18 for GPT-4 (20).

### 3.5 NLP in medical literature mining

The rapidly expanding volume of medical literature poses significant challenges for healthcare professionals attempting to stay abreast of the latest developments and establish meaningful connections between findings. Despite numerous resources for searching medical literature, the ability to distill and translate knowledge into actionable patient care insights remains predominantly available through paid services.

The increase in open-access articles has amplified the volume of information available for review and added to the complexity of synthesizing this data effectively. There is a pressing need for intelligent systems capable of searching literature using advanced keyword strategies, constructing meaningful semantic connections between publications, and extracting and assessing knowledge tailored to specific patient needs.

State-of-the-art models addressing these challenges include those trained in medical comprehension, such as BioBERT, and systems leveraging Retrieval-Augmented Generation (RAG). These advanced models utilize search embeddings to process large volumes of documents, retrieve relevant information, and perform summarization linked to individual patient scenarios. By integrating such capabilities, these AI systems demonstrate the potential for enhancing the accessibility and usability of medical knowledge in clinical practice.

The current landscape of medical literature is dynamic and increasingly accessible, underscoring the importance of innovative tools to bridge the gap between knowledge acquisition and application in patient care (21).

### 3.6 Genomics

The advent of next-generation sequencing (NGS) and the subsequent genome human genome mapping have catalyzed an explosion in medical knowledge, ushering in the era of truly massive data. These advancements have facilitated discoveries previously beyond imagination (22). Complex computational models are now essential for identifying genetic mutations, copy number variations, structural anomalies, quantitative changes in RNA expression (transcriptomics), and qualitative and quantitative alterations in methylation and other epigenetic modifications.

Recently, the groundbreaking determination of 3D protein structures from amino acid sequences, recognized with a Nobel Prize, has opened unprecedented opportunities for cancer research. This breakthrough promises numerous discoveries that are poised to improve patient outcomes. Concurrently, second-generation phenotyping, which analyzes subtle facial and physical characteristics, is advancing the prediction of genetic abnormalities with remarkable precision.

In pediatric research, liquid biopsy techniques utilizing circulating tumor DNA (ctDNA) are gaining traction, enabling minimally invasive diagnostics and monitoring (23). The study of somatic mutations (acquired within tumors) and germline mutations (inherited or constitutional) has become pivotal in pharmacogenomics and biomarker discovery, driving personalized medicine and advancing pediatric oncology practice (24, 25).

Large language models (LLMs) are used to analyze large volumes of text data, such as medical records and research papers, to extract meaningful insights and support clinical decision-making. For example, Rady Children’s Hospital and the Institute for Genomic Medicine are building pediatric genomic LLMs to improve the treatment of rare pediatric diseases. LLMs can help researchers and clinicians access and interpret vast amounts of information, potentially leading to faster diagnoses and more effective treatments (26).

Generative AI techniques are used to create new data that resembles accurate data, which can augment existing datasets or train AI models in situations where data is scarce. This is particularly valuable in pediatric oncology, where data can be limited due to the rarity of childhood cancers. The Elizabeth Glaser Pediatric AIDS Foundation is applying generative AI techniques to provide clinicians with real-time insights into HIV patient risks in children. By generating synthetic data, researchers can overcome data limitations and develop more robust AI models.

ML and generative AI models are used to analyze complex datasets and make predictions, such as predicting treatment responses or identifying new biomarkers. Memorial Sloan Kettering Cancer Center is developing advanced ML and generative AI models to drive improvements in translational research and broad-scale changes in precision cancer care for children worldwide. These models can help identify patterns and insights that may not be apparent to human observers, leading to more personalized and effective treatments (27).

Deep learning approaches for single-cell analysis are also being explored. Researchers like Dr. Fabian are using deep learning, a subfield of machine learning, to model single-cell variation and understand cellular responses, potentially leading to the development of single-cell foundation models. This research could lead to a deeper understanding of the complex interactions within tumors and pave the way for new therapeutic strategies.

Projects like THALES leverage ML frameworks to analyze family genetic data and predict cancer risk, enabling early intervention strategies. AI supports cancer subtyping, mutation prediction, and prognostication by integrating multi-omics data, including genomics, radiomics, and transcriptomics. For instance, AI models have been developed to classify pediatric brain tumors into clinically significant subgroups, aiding in tailored treatment planning.

Despite these advancements, challenges remain, including the need for large, high-quality datasets, and the complexity of integrating diverse data types. Ethical considerations, such as data privacy and the potential for genetic discrimination, must also be addressed to ensure the responsible use of AI in genomic analysis.

## 4 Artificial intelligence in pediatric oncology

Despite the current AI advancements, the development of sustainable AI tools depends upon the availability of large datasets with strict quality control. Several biomedical imaging repositories have been created to date, such as The Cancer Imaging Archive (TCIA), focusing on cancer imaging, and PRIMAGE project, an open cloud-based platform based on the European population with high-quality anonymized datasets (imaging, clinical, molecular, and genetics) (28). Although they have huge potential, these repositories have been created as stand-alone entities. As such, we still need repositories that are fully findable, accessible, interoperable, and reusable repository based on multiple populations for AI analysis (29).

The development of AI techniques promotes numerous potential applications in pediatric oncology (30). AI algorithms provide timely references based on large amounts of data. In contrast, DL-CNNs can learn automatically from medical literature that can assist in precise diagnosis, which can assist in correct diagnosis and optimal treatment selection. The present review provides insights into emerging AI applications for pediatric oncology (31, 32).

### 4.1 Diagnostic accuracy and early detection

AI systems can identify patterns in medical images that may indicate cancerous growth. It has been successfully applied to various pediatric cancers. For instance, studies have shown that AI can achieve diagnostic accuracy rates exceeding 90% for conditions like acute lymphoblastic leukemia (ALL) and other hematological malignancies by analyzing microscopic images and patient histories (30, 33, 34). Another example is intracranial tumors, where ML models have improved diagnostic accuracy for brain tumors by analyzing MRI sequences, particularly for challenging cases where tumor types overlap (35).

Artificial Intelligence (AI)-driven radiomics has emerged as a powerful approach to clinical imaging in pediatric oncology. Radiomics involves extracting large amounts of quantitative data from medical images, offering enhanced predictive capabilities. Recent studies have shown significant promise in using CT-based radiomic features to predict pulmonary metastasis risk in pediatric osteosarcoma patients at initial diagnosis (14). Additionally, multimodal imaging that combines PET and MRI, enhanced by AI methodologies, has improved the accuracy of detecting pediatric lymphoma, illustrating the advantage of integrating functional and anatomical data (33). Furthermore, MRI-based radiomic signatures have successfully differentiated molecular subtypes of pediatric medulloblastoma, providing a valuable non-invasive diagnostic tool (36). The inclusion of these methodologies—radiomics, CT, PET, and multimodal imaging—represents significant progress toward precision diagnosis in pediatric oncology.

### 4.2 Predictive analytics

Predictive analytics involves using statistical algorithms and AI techniques to analyze historical data and predict future events. In pediatric oncology, predictive analytics can forecast treatment responses, disease progression, and patient outcomes, supporting personalized treatment planning and improving clinical decision-making.

Personalized treatment planning harnesses AI-driven predictive analytics to optimize therapy tailored to individual patient profiles. For instance, machine learning models have been effectively utilized to forecast chemotherapy-induced toxicities, such as severe myelosuppression in pediatric Wilms tumor patients, thereby allowing clinicians to adjust chemotherapy dosing and supportive care proactively (12). Moreover, AI algorithms that integrate multi-modal patient data—including genomic, radiomic, and clinical factors—have proven instrumental in stratifying patients into risk groups, enabling personalized therapeutic approaches that enhance both efficacy and safety (28, 32). Additionally, AI-powered auto-segmentation tools have improved the precision and efficiency of radiotherapy planning in pediatric oncology by accurately delineating tumors and organs-at-risk, reducing human error, and improving treatment outcomes (27).

Ramesh et al. reviewed the application of AI in pediatric oncology. They elegantly listed recent literature and highlighted multiple examples of the use of AI in predicting features and outcomes of pediatric cancer (37).

#### 4.2.1 CNS tumors

1. *Tumor classification using neural networks:*
  ◦ Quon et al. applied a neural network model to classify posterior fossa tumors in pediatric CNS patients using 816 MRI images. The model achieved an AUROC of 0.99, classification accuracy of 92%, and sensitivity and specificity of 0.96 and 1.00, respectively (38).
2. *Medulloblastoma subtype classification*:
  ◦ Iv et al. used a support vector machine (SVM) model on MRI data to classify medulloblastoma into molecular subgroups (e.g., Sonic Hedgehog, Group 3, and Group 4), achieving AUROCs of 0.79, 0.70, and 0.83, respectively. However, the model struggled with the Wingless subtype (AUROC = 0.45) (36, 39).
3. *Differentiating tumor types with SVM*
  ◦ Fetit et al. utilized an SVM model trained on 3D textural attributes of MRI scans to differentiate medulloblastomas, ependymomas, and pilocytic astrocytomas. AUROCs ranged from 0.76 to 0.86 in pairwise testing (40, 41).

#### 4.2.2 Extracranial solid tumors

4. *Necrosis prediction in osteosarcoma*
  ◦ Huang et al. developed a random forest model to identify regions of necrosis in histology slides of osteosarcoma patients post-chemotherapy, achieving a sensitivity of 94%, specificity of 78%, and AUROC of 0.90 (42).
5. *Pathology slide analysis for tumor necrosis*
  ◦ Arunachalam et al. used both SVM and deep learning models to predict tumor necrosis in osteosarcoma slides, each achieving an AUROC of 0.99 (43).
6. *Ewing sarcoma diagnosis*
  ◦ Chaber et al. employed a quadratic discriminant analysis classifier on MRI scans to distinguish Ewing sarcoma from osteomyelitis, achieving precision and recall metrics of 75-88% (44).
7. Neuroblastoma histologic classification
  ◦ Gheisari et al. used a Convolutional Deep Belief Network on 1,043 histologic images to classify neuroblastoma subtypes with a precision of 84.5% and recall of 87.6% (45).

#### 4.2.3 Leukemia

8. *Predicting relapse in ALL*
  ◦ Pan et al. developed a random forest model to predict relapse in pre–B-ALL patients, achieving an AUROC of 0.904. This study incorporated external validation with an 84-patient cohort (46).
9. *Cognitive impairment post-chemotherapy*
  ◦ Kesler et al. used a random forest model combining MRI and demographic data to predict cognitive impairment in ALL survivors, achieving an accuracy of 89.4%, sensitivity of 95.8%, and specificity of 85.7% (47).
10. *Detection of minimal residual disease*
  ◦ Reiter et al. utilized a Gaussian Mixture Model to detect MRD in bone marrow samples from B-ALL patients, with a precision of 0.81, recall of 0.90, and F1 score of 0.80 (48).

While these models all work well in an experimental model, a question remains whether their results can be generalized to more subjects. Variations in collecting and processing features and heterogeneity in patients’ populations, as well as heterogeneity in patients’ populations, make some models of little value in clinical practice, hence the need for real-world data to validate these models.

### 4.3 Drug development for pediatric cancers

Artificial intelligence is significantly accelerating drug discovery and pharmaceutical development in pediatric oncology. AI-based platforms facilitate rapid *in silico* screening of vast chemical libraries, swiftly identifying potential drug candidates effective against specific pediatric cancer targets (49). Additionally, machine learning algorithms analyze genomic and proteomic datasets to identify novel therapeutic targets specific to pediatric malignancies, guiding targeted therapy development (49). Groundbreaking AI applications such as deep-learning-driven protein structure prediction tools, notably AlphaFold, have also opened new avenues for drug development, enabling structure-guided therapeutic design previously unattainable in pediatric oncology (50). These advancements promise more efficient, targeted, and effective treatment options for children affected by cancer.

### 4.4 Expanded AI tools and software

Numerous advanced AI tools and platforms are now accessible, significantly benefiting pediatric oncology. Resources like The Cancer Imaging Archive (TCIA) and the PRIMAGE platform provide extensive repositories of anonymized pediatric cancer imaging data, facilitating the development and validation of AI models (51). Clinical data management has been streamlined by automated software such as ExtractEHR, enabling efficient extraction and analysis of electronic health records (EHRs) specifically tailored for pediatric oncology (19). Moreover, tools like PRISM, which utilizes specialized large language models (LLMs), are revolutionizing patient recruitment by automatically matching patient records with relevant clinical trials (20). At institutions such as Rady Children’s Hospital, specialized pediatric genomic LLMs are being developed to interpret complex genetic data rapidly and accurately, supporting clinical decision-making in rare pediatric diseases (26). The availability and integration of these sophisticated tools mark significant progress toward comprehensive, AI-enabled pediatric cancer care.

## 5 Ethical issues associated with the use of AI in pediatric oncology

AI currently represents a technology sector with one of the strongest, most substantial, and most vigorous growths. Although initially considered just another technology that could be applied to the medical world, it was quickly realized that AI has different characteristics from most other technologies due to its potential to replace human reasoning and decision-making. As a result, debate quickly arose regarding its use. The process of understanding and accepting AI is dynamic, and driven, on the one hand, by changes in the level of knowledge about technology and, on the other, by its exponentially growing evolution. The shared opinion is that people’s perception of AI depends on contextual factors rather than on the underlying algorithm (52).

Barriers and enablers for adopting AI innovations have been widely studied across many medical fields, with radiology and oncology frequently addressing perception issues (49, 53). One study revealed that while the overall attitude toward AI was generally positive, a significant concern—especially in pediatric applications—was the potential for error. Additionally, significant differences in AI perception were observed among groups defined by education level (54).

To ensure fundamental ethical principles are prioritized through the ideation, development, deployment, and evaluation of AI and ML studies, researchers have highlighted the importance of formal ethics reviews to improve safety and promote equity. If best practice standards are not established for children, the rapid expansion of AI research can potentially widen existing gaps (55, 56). As the research community develops consensus guidelines for AI algorithms and refines the ethical use of AI, as well as particular protections for the pediatric population, are essential. One of the frameworks that was proposed, ACCEPT-AI, which is a set of principles and key recommendations that can be used independently or flexibly embedded in existing and emerging future guidelines (57).

## 6 Conclusion

Precision medicine has ushered in significant advances in diagnosing and treating pediatric cancers. The growing use of technologies like NGS has facilitated genomic landscape and functional characterization studies, enhancing our understanding of cancer biology and improving the classification and risk stratification of numerous cancers. By leveraging large-scale genomic data and real-world clinical records, AI-powered tools are revolutionizing cancer diagnosis, predicting treatment outcomes, and guiding individualized therapeutic strategies. This advancement not only enhances diagnostic accuracy but also minimizes treatment-related toxicities and improves survival rates in pediatric cancer patients.

Our review highlights the potential of AI models to bridge the gap between complex biological data and clinical decision-making, paving the way for more effective and tailored interventions. However, despite significant progress, challenges such as data standardization, ethical considerations, and model interpretability remain. Future efforts should focus on enhancing AI algorithms’ transparency and ensuring equitable access to AI-driven solutions across diverse patient populations.

AI is promising to transform pediatric oncology through enhanced diagnostic capabilities and personalized treatment approaches. Continued research and collaboration among clinicians, researchers, and technologists are crucial to overcoming current challenges and unlocking the full potential of AI in advancing precision medicine for children with cancer.
